# Exploring the Mechanism of Asiatic Acid against Atherosclerosis Based on Molecular Docking, Molecular Dynamics, and Experimental Verification

**DOI:** 10.3390/ph17070969

**Published:** 2024-07-22

**Authors:** Zhihao Wu, Luyin Yang, Rong Wang, Jie Yang, Pan Liang, Wei Ren, Hong Yu

**Affiliations:** 1 School of Basic Medical Sciences, Southwest Medical University, Luzhou 646000, China; wzhkycg@163.com (Z.W.); swmu_wangrong@163.com (R.W.);; 2 National Traditional Chinese Medicine Clinical Research Base and Drug Research Center of Integrated Traditional Chinese and Western Medicine, the Affiliated Traditional Chinese Medicine Hospital, Southwest Medical University, Luzhou 646000, China; yangly2021@swmu.edu.cn (L.Y.); xnydzyylp@swmu.edu.cn (P.L.); 3 Institute of Integrated Chinese and Western Medicine, Southwest Medical University, Luzhou 646000, China; 4 Public Center of Experimental Technology, Southwest Medical University, Luzhou 646000, China

**Keywords:** asiatic acid, atherosclerosis, PPARγ/NF-κB signaling pathway

## Abstract

Asiatic acid (AA) is a pentacyclic triterpene derived from the traditional medicine *Centella asiatica*. It is known for its anti-inflammatory, antioxidant, and lipid-regulating properties. Though previous studies have suggested its potential therapeutic benefits for atherosclerosis, its pharmacological mechanism is unclear. The objective of this study was to investigate the molecular mechanism of AA in the treatment of atherosclerosis. Therefore, network pharmacology was employed to uncover the mechanism by which AA acts as an anti-atherosclerotic agent. Furthermore, molecular docking, molecular dynamics (MD) simulation, and in vitro experiments were performed to elucidate the mechanism of AA’s anti-atherosclerotic effects. Molecular docking analysis demonstrated a strong affinity between AA and PPARγ. Further MD simulations demonstrated the favorable stability of AA-PPARγ protein complexes. In vitro experiments demonstrated that AA can dose-dependently inhibit the expression of inflammatory factors induced by lipopolysaccharide (LPS) in RAW264.7 cells. This effect may be mediated through the PPARγ/NF-κB signaling pathway. This research underscores anti-inflammation as a crucial biological process in AA treatments for atherosclerosis, with PPARγ potentially serving as a key target.

## 1. Introduction

Atherosclerosis (AS) is the main factor underlying cardiovascular disorders, characterized by the accumulation of lipids, the formation of plaques, and a narrowing of arterial lumens [1]. The oxidation of lipid particles that accumulate beneath the arterial endothelium, particularly low-density lipoprotein cholesterol (LDL-C), is believed to be the initial step in the formation of AS plaques [2]. Oxidized low-density lipoprotein (ox-LDL) can trigger the release of various cytokines and inflammatory mediators, leading to an elevated production of pro-inflammatory mediators and thereby exacerbating inflammation [3]. Subsequent to the endothelial inflammatory response, circulating monocytes attach to the endothelium and migrate into the subendothelial area [4]. Monocytes/macrophages undergo transformations into foam cells under the influence of ox-LDL [5]. In advanced lesions, excess free cholesterol results in the death of foam cells and the formation of a necrotic core prone to rupture. Recent clinical trials have demonstrated that anti-inflammatory medicines can significantly reduce the risk of cardiovascular events in people with AS [6]. Compared with synthetic compounds, natural products have good therapeutic effects and safety [7]. Crucially, natural products are often demonstrated to have strong anti-inflammatory capabilities and are considered to be safer solutions compared to synthetic compounds. This indicates that they could be viable options for treating AS [8].

Asiatic acid (AA), a pentacyclic triterpenoid isolated from the traditional medicinal plant *Centella asiatica* (*C. asiatica*), demonstrates diverse pharmacological properties such as anti-inflammation, antioxidant, and cardiovascular protection activities [9]. AA has attracted significant attention due to its potent anti-inflammatory effects. Research indicates that AA can improve diabetic retinopathy by inhibiting the NF-κB signaling pathway to modulate the polarization of microglial cells [10]. Additionally, AA shows potential in alleviating inflammation-related conditions such as acute lung injury [10,11], liver fibrosis [12], and Alzheimer’s disease [13] by suppressing pro-inflammatory cytokines. Recent studies suggest that AA’s anti-atherosclerotic effects are linked to its protective actions on the vascular endothelial barrier [14], lipid regulation [15], and the correction of hemodynamic abnormalities [16]. These findings highlight the multifaceted mechanisms through which AA exerts its anti-atherosclerotic effects; however, the exact mechanism is unclear. Therefore, we attempted to investigate the anti-inflammatory mechanism of AA, especially the inhibitory inflammatory factors it may target.

Network pharmacology is a systematic approach that anticipates the relationship between chemicals and disease targets [17]. This approach is valuable in identifying key targets relevant to drugs and diseases. Molecular docking is a useful tool for predicting and designing new drugs that allows exploration of the binding modes of component–target interactions [18]. Molecular dynamics (MD) simulations can be used to estimate the structural stability of receptors and ligands, as well as the dynamics of receptor–ligand interactions using appropriate force fields [19]. These methods are commonly employed in natural product development research [20,21]. In this study, network pharmacology was used in conjunction with molecular docking and MD simulations to predict and verify the potential target of AA in treating AS. Additionally, the therapeutic mechanism of AA on AS was verified through an in vitro experiment.

## 2. Results

### 2.1. Intersection Targets of AA- and AS-Related Targets

**Figure 1:**
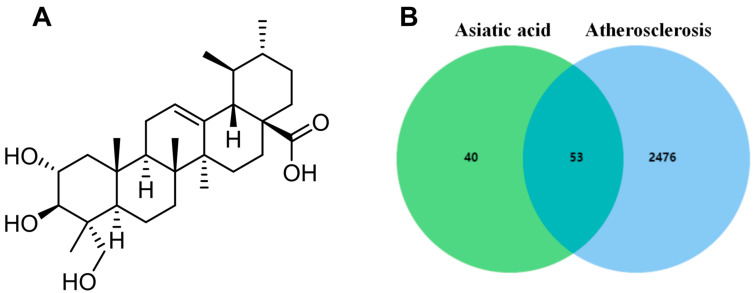
Network pharmacology analysis: (**A**) chemical structure of AA and (**B**) Venn diagram of potential targets.

The chemical structure of AA is shown in Figure 1A, and its predicted targets were obtained from the HERB, ETCM, and Swisstargetprediction databases. Then, the results of the three databases were summarized and duplicate data were deleted, obtaining a total of 93 drug targets. In the GeneCards database, 2529 disease-related targets were searched using the keyword “atherosclerosis”. Finally, drug and disease targets were uploaded to Venn diagram analysis, resulting in a total of 53 common targets (Figure 1B).

### 2.2. Construction and Analysis of the Protein–Protein Interaction (PPI) Network

**Figure 2:**
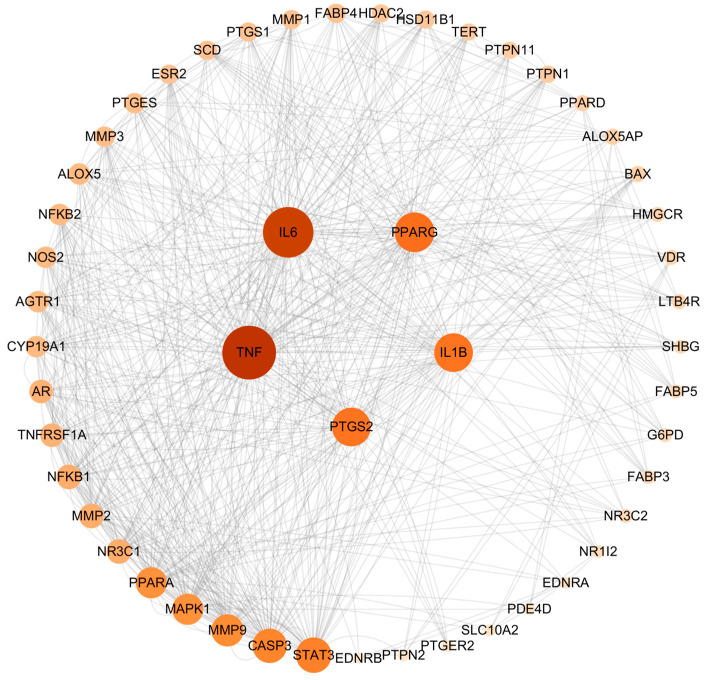
PPI network of common targets.

The intersection targets were imported into the String database for PPI analysis. The network included 53 nodes and 376 edges. The results were then analyzed using the cytoHuba plug-in of Cytoscape 3.10.0 software, and the degree value was calculated. The higher the value of the degree, the more important the target. The top five targets of AA against AS were identified as TNF, IL-6, PPARγ, PTGS2, and IL-1β (Figure 2).

### 2.3. Biological Function Enrichment Analysis

**Figure 3:**
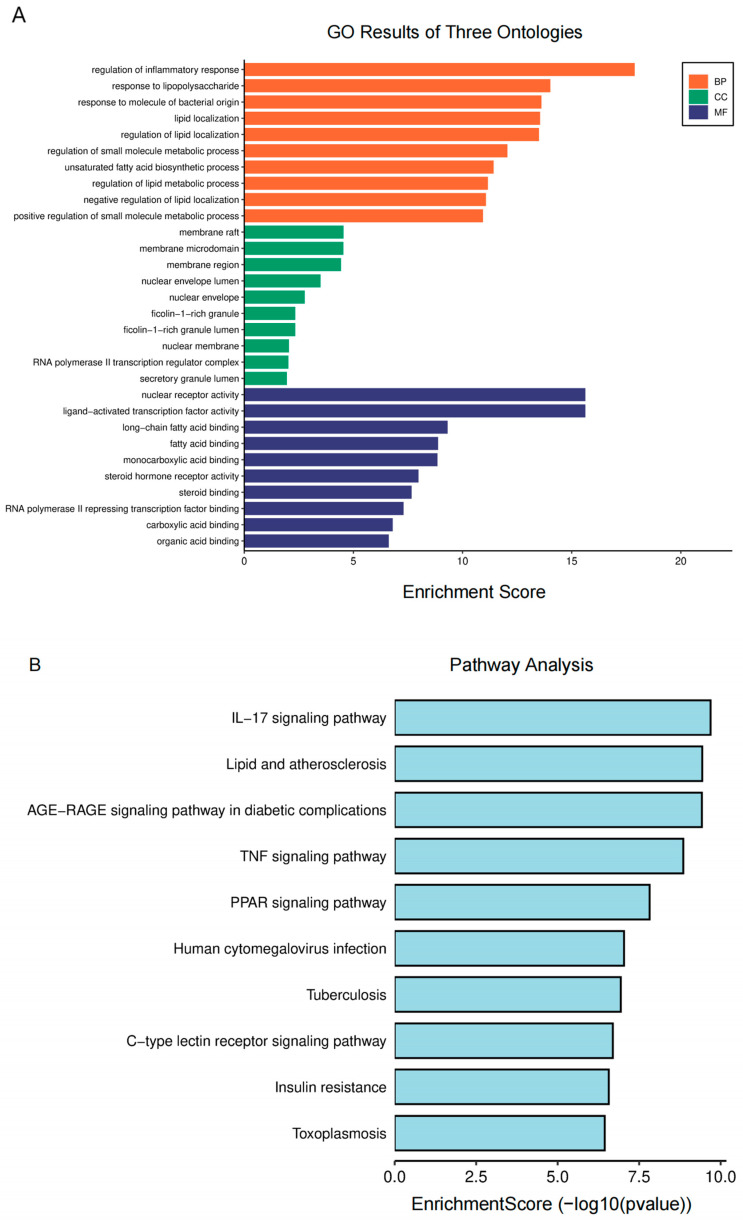
Biological function enrichment analysis of AA treatment: (**A**) GO analysis of AA targets and (**B**) KEGG pathway analysis of AA targets.

For further analysis, GO and KEGG enrichment analyses were performed. The GO enrichment terms encompass biological process (BP), cellular component (CC), and molecular function (MF). The analysis of BP suggests that the regulation of inflammatory and lipopolysaccharide responses plays a crucial role in AA treatment for AS (Figure 3A). MF was primarily associated with the nuclear receptor, ligand-activated transcription factor, long-chain fatty acid binding, and fatty acid binding activities. Additionally, CC primarily consisted of membrane rafts, microdomains, and regions. According to the KEGG research findings (Figure 3B), the pathways of AA for the treatment of AS primarily involve lipid metabolism-related signals (lipids and atherosclerosis and PPARγ signaling pathways) and inflammatory response-related signals (IL-17 and TNF signaling pathways). These findings suggest that AA can exert anti-AS effects through multiple pathways.

### 2.4. Molecular Docking

Molecular docking of AA was performed with core targets (TNF, IL6, PPARγ, PTGS2, and IL1β) in order to investigate its binding mode and affinity. The binding free energy in molecular docking is a crucial factor in assessing the stability of drug–target interactions. Generally, a lower binding energy indicates a more stable interaction between the ligand and the receptor. Binding energy values below 0 indicate spontaneous binding between the ligand and the receptor, while values ≤ −7.0 kcal/mol indicate a good binding ability [22,23].

**Figure 4:**
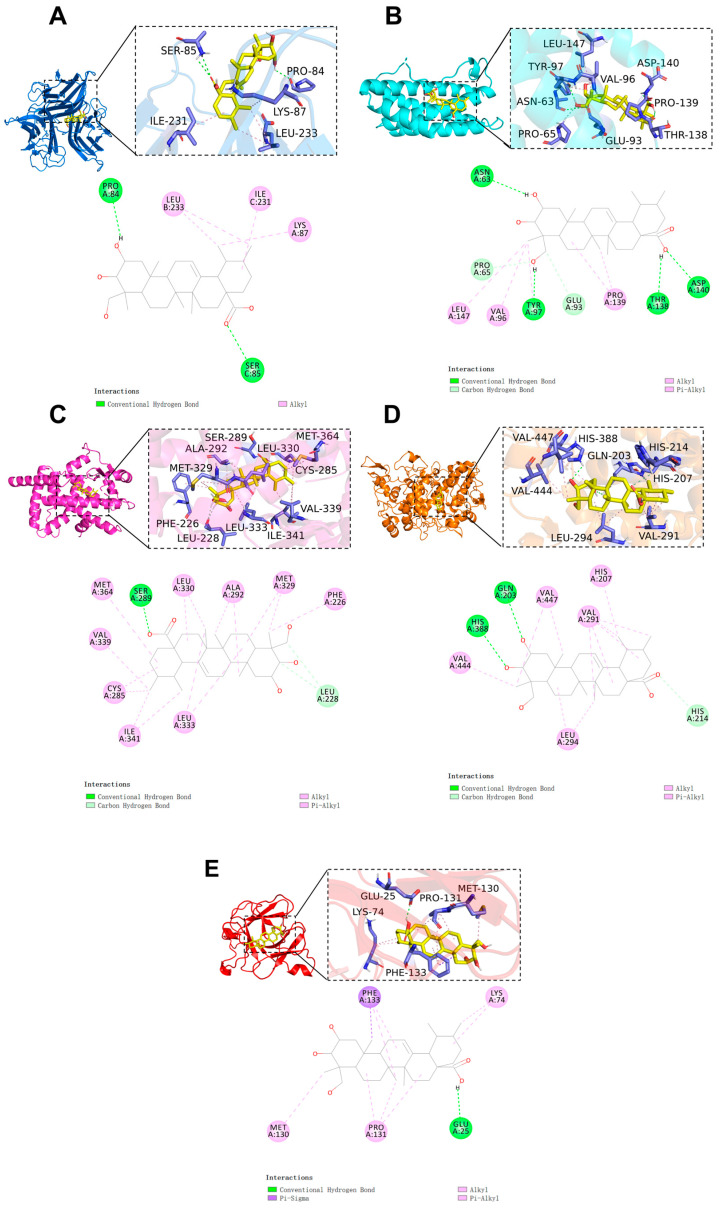
Molecular docking results of AA and core targets: (**A**) TNF-α, (**B**) IL-6, (**C**) PPARγ, (**D**) PTGS2, and (**E**) IL-1β.

The binding configuration and the interaction site of AA with the core target are shown in Figure 4. The results indicate that the binding free energy between AA and the core targets is less than −6 kcal/mol (AA-TNF complex, binding energy: −6.3 kcal/mol; AA-IL6 complex, binding energy: −6.9 kcal/mol; AA-PPARγ complex, binding energy: −9.3 kcal/mol; AA-PTGS2 complex, binding energy: −6.9 kcal/mol; AA-IL1β complex, binding energy: −7.0 kcal/mol). This indicates that the compound has a high binding affinity for the target. Notably, PPARγ exhibited the lowest binding free energy. The results show that AA and the PPARγ protein form hydrogen bonds with the SER 289 residue and interact with nine alkyl and π-alkyl groups, including LEU 333, ILE 341, CYS 285, VAL 339, MET 364, LEU 330, ALA 292, and MET 329. Additionally, carbon–hydrogen bonds were detected in LEU 228. All of these interactions contribute to the stability of the complex (Figure 4C).

To validate the docking result, the cocrystal ligand VSP-77 was redocked. The molecular docking result was deemed reliable if the root mean square deviation (RMSD) value was less than 2.5. The results show that the corresponding RMSD value is 1.127 Å. This indicates that the molecular docking method is reliable.

### 2.5. Molecular Dynamics

The molecular docking results demonstrate that AA exhibits strong binding affinity with the key target PPARγ protein. Consequently, MD simulations were conducted on the complexes of AA-PPARγ and VSP-77-PPARγ.

**Figure 5:**
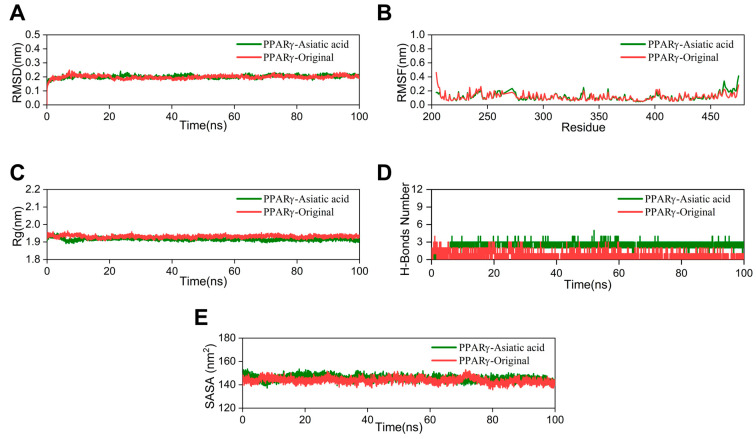
MD simulation: (**A**) RMSD curves, (**B**) RMSF curves, (**C**) Rg curves, (**D**) H-bonds plot, and (**E**) SASA plot.

The RMSD value indicates the stability of the ligand–protein complex. The lower the RMSD value, the greater the stability [24]. The RMSD curve of the AA group nearly overlaps with that of the crystal ligand, with a fluctuation range of around 0.2 nm (Figure 5A). Root mean square fluctuation (RMSF) is utilized to demonstrate fluctuations in the complex at the residue level [25]. The RMSF curve of the AA-PPARγ protein complex shows minimal fluctuations. Only the amino acid residues at positions 460–470, at the periphery of the protein structure, demonstrate a moderate variation of approximately 0.5 nm (Figure 5B). The radius of gyration (Rg) was utilized to assess the compactness and stability of the structure. A smaller Rg value suggests that the system maintains its compactness and stability [26]. As shown in Figure 5C, the Rg curve of the AA-PPARγ protein complex fluctuates within a range of 2 nm throughout the process and nearly completely overlaps with the cocrystal ligand. The number of hydrogen bonds serves as an important indicator in MD simulations. These hydrogen bond interactions are crucial for maintaining the stability of protein–ligand complexes [27]. The results indicate that the number of hydrogen bonds in the AA-PPARγ complex stabilizes at around three after 5 ns and does not exhibit significant fluctuations. The number of hydrogen bonds between AA and PPARγ is significantly higher than in the cocrystal ligand, ranging from 1 to 3 and exhibiting significant fluctuations (Figure 5D). In MD simulations, the solvent-accessible surface area (SASA) is a key parameter for determining the folding and stability of proteins. A more stable SASA curve generally indicates a more stable protein structure [28]. The SASA curve of the PPARγ protein–AA complex fluctuated stably throughout the process without obvious deviations (Figure 5E).

**Figure 6:**
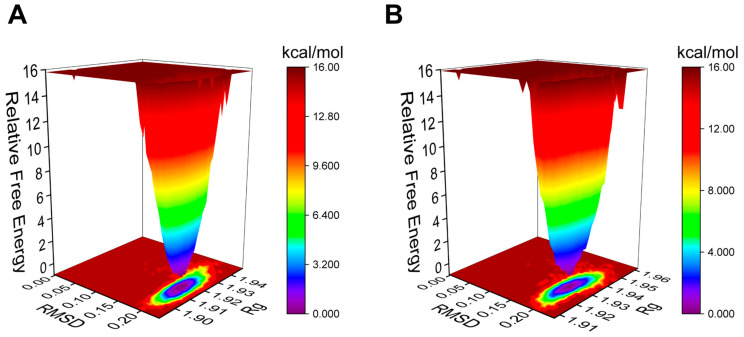
Plots of Gibbs FEL: (**A**) AA-PPARγ and (**B**) VSP-77-PPARγ.

The Gibbs free energy landscape (FEL) is employed to characterize the lowest energy configuration during simulations of complicated structural dynamics [29]. Dark purple/blue spots denote the lowest energy values and the most stable structures, while unstable structures are represented by red/yellow dots. The Gibbs FEL of the AA-PPARγ protein complex displays a concentrated minimum energy cluster and a relatively concentrated energy distribution, similar to the cocrystal ligand (Figure 6A,B). This indicates that the complex formed by the AA-PPARγ protein has good stability. 

**Figure 7:**
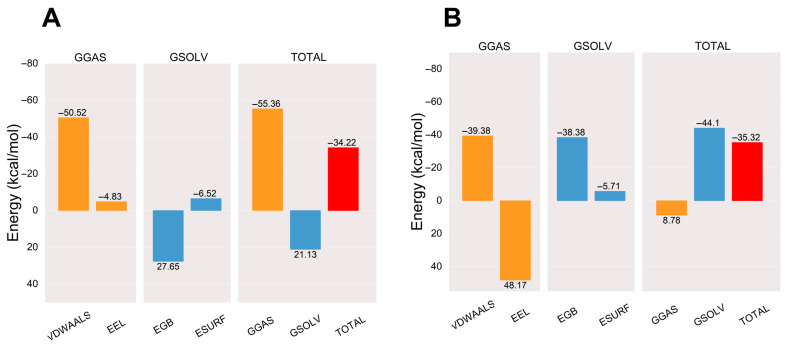
Plots of MM/GBSA binding energy: (**A**) AA-PPARγ and (**B**) VSP-77-PPARγ. VDWAALS, EEL, EGB, ESURF, GGAS, GSOLV, and TOTAL represent Van der Waals forces, electrostatic energy, polar solvation energy, nonpolar solvation energy, molecular mechanics terms, solvation energy terms, and average binding free energy, respectively.

After the complex system has stabilized, the molecular mechanics/generalized Born surface area (MM/GBSA) binding energy of the protein–ligand complex is calculated [30]. The average binding free energy of the AA-PPARγ protein complex is −34.22 kcal/mol, which is comparable to the average binding free energy with the cocrystal ligand (−35.32 kcal/mol). The binding energies E_VDWAALS_, E_EEL_, E_EGB_, E_ESURF_, E_GGAS_, and E_GSOLV_ between AA and PPARγ were −50.52, −4.83, 27.65, −6.52, −55.36, and 21.13 kcal/mol, respectively. The results show that the affinity of the AA-PPARγ protein complex has a strong binding affinity. The results are shown in Figure 7.

### 2.6. Inhibition of Nitric Oxide (NO) Secretion in RAW264.7 Cells 

**Figure 8:**
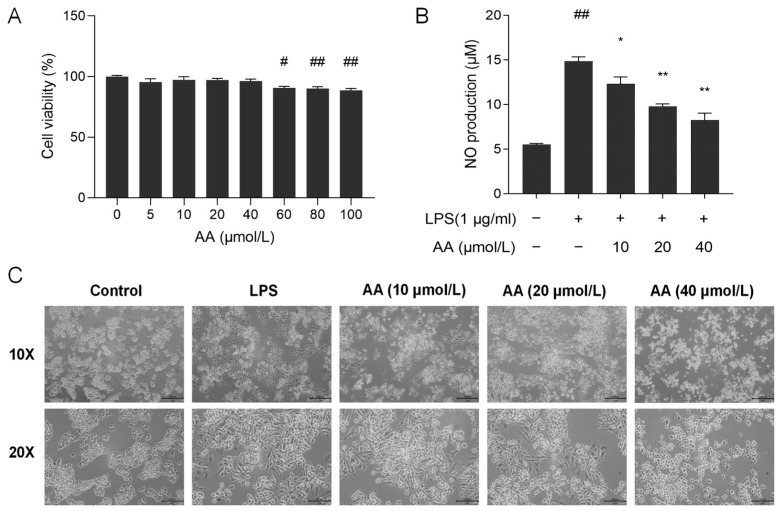
(**A**) Cell viability after treatment with AA. (**B**) Effect of AA on LPS-induced NO secretion. (**C**) Effect of AA on the morphology of cells. The results are presented as mean values ± standard error of the mean (SEM), with statistical significance indicated at a *p*-value below 0.05 (compared with the control group, # *p* < 0.05, ## *p* < 0.01; compared with LPS group, * *p* < 0.05, ** *p* < 0.01).

To evaluate the effect of AA on the viability of RAW264.7 cells, the CCK8 assay was performed. As shown in Figure 8A, AA did not show cytotoxic effects on RAW264.7 cells at concentrations below 40 µmol/L. Therefore, the concentrations of 10, 20, and 40 µmol/L AA were selected for further investigation.

NO serves as a crucial biomarker in the inflammatory process, with excessive NO release capable of exacerbating the inflammatory response [31]. As shown in Figure 8B, lipopolysaccharide (LPS) stimulation resulted in a significant increase in the secretion of NO in the supernatant (*p* < 0.01) compared with the control group. Following pretreatment with AA, a notable decrease in NO secretion in the cell supernatant was observed (*p* < 0.05). Our results showed that AA dose-dependently inhibits NO production.

### 2.7. Cell Morphology

The cell morphology of RAW264.7 was examined using an inverted microscope. Cells in the control group had a round and plump appearance (Figure 8C). After LPS induction, the cell shape was irregular and rough, the cells expanded rapidly, pseudopodia were expanded, and the cytoplasm was highly vacuolated. Pseudopodia were reduced in AA-treated cells, and most cells appeared round. The alterations in cell morphology became more pronounced with increasing concentrations of AA.

### 2.8. Effects of AA on LPS-Induced Reactive Oxygen Species (ROS) Generation

**Figure 9:**
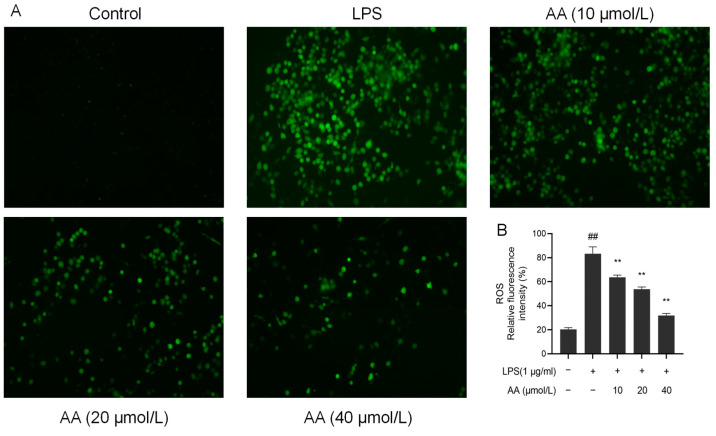
AA inhibited the LPS-induced generation of ROS in RAW264.7 cells: (**A**) fluorescence microscopy images and (**B**) intracellular relative ROS levels. The results are presented as mean values ± standard error of the mean (SEM), with statistical significance indicated at a *p*-value below 0.05 (compared with the control group, ## *p* < 0.01; compared with LPS group, ** *p* < 0.01).

Upon LPS stimulation, inflammatory cells release a variety of ROS, activating inflammatory signaling pathways and leading to cell and tissue damage [32]. There was a significant increase in the fluorescence intensity of ROS in the model group (*p* < 0.05, Figure 9). Notably, pretreatment with AA for 2 h resulted in a significant dose-dependent reduction in ROS production (*p* < 0.05).

### 2.9. Effects of AA on the Expression of TNF-α, IL-6, iNOS, COX-2, and Arg-1 in LPS-Stimulated RAW264.7 Cells

**Figure 10:**
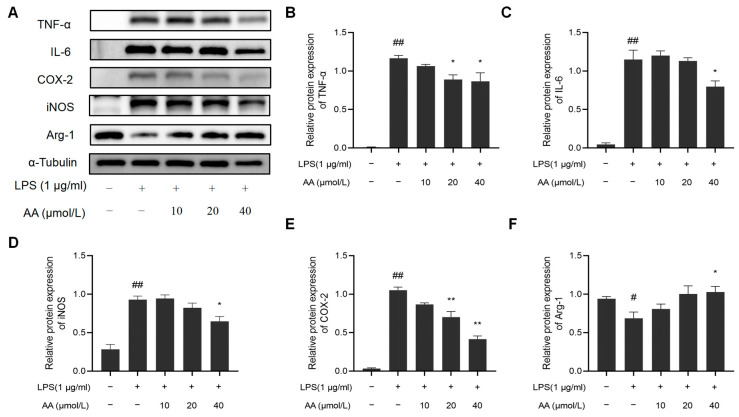
Effect of AA on inflammatory factor expression: (**A**) Western blot results, (**B**) TNF-α, (**C**) IL-6, (**D**) COX-2, (**E**) iNOS, and (**F**) Arg-1. The results are presented as mean values ± standard error of the mean (SEM), with statistical significance indicated at a *p*-value below 0.05 (compared with the control group, # *p* < 0.05, ## *p* < 0.01; compared with LPS group, * *p* < 0.05, ** *p* < 0.01).

In addition, we examined whether AA suppresses the inflammatory response in LPS-stimulated RAW264.7 cells. LPS notably increased the protein expression of COX-2, iNOS, TNF-α, and IL-6 while it inhibited the protein expression of Arg-1. Remarkably, the AA (40 µmol/L) group showed a significant reduction in the protein expression of COX-2, iNOS, TNF-α, and IL-6 compared to the model group and a significant increase in Arg-1 expression (*p* < 0.05). The results are shown in Figure 10.

### 2.10. The Effect of AA on NF-κB Inflammatory Signaling Pathway in RAW264.7 Cells

**Figure 11:**
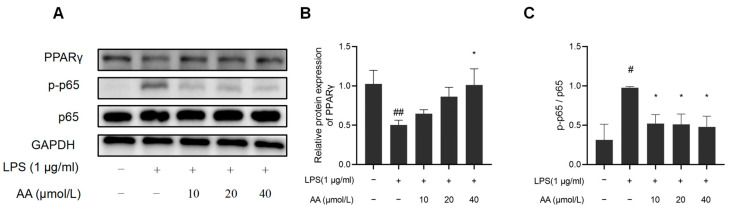
Effects of AA on NF-κB activation and PPARγ expression: (**A**) Western blot results, (**B**) NF-κB p65, p-p65 protein expression levels, and (**C**) PPARγ protein expression levels. The results are presented as mean values ± standard error of the mean (SEM), with statistical significance indicated at a *p*-value below 0.05 (compared with the control group, # *p* < 0.05, ## *p* < 0.01; compared with LPS group, * *p* < 0.05).

NF-κB activation and PPARγ expression were further investigated to elucidate the potential anti-inflammatory properties of AA (Figure 11). The results indicated that LPS significantly reduced PPARγ protein expression, while the protein expression of pho NF-κB p65 was observed to increase, suggesting activation of the NF-κB pathway (*p* < 0.05). AA treatment significantly inhibited LPS-induced NF-κB activation, and the PPARγ expression significantly increased (*p* < 0.05).

### 2.11. The Effect of AA on LPS-Stimulated RAW264.7 Cell Inflammation May Be PPARγ-Dependent

**Figure 12:**
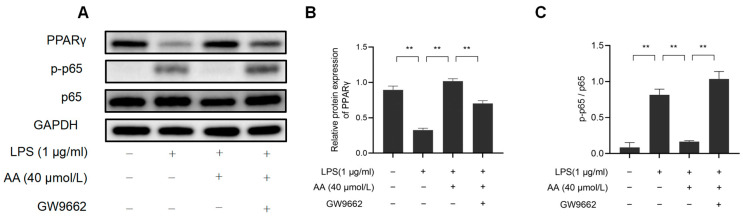
AA regulates macrophage inflammation through the PPARγ/NF-κB signaling pathway: (**A**) Western blot results, (**B**) NF-κB p65, p-p65 protein expression levels, and (**C**) PPARγ protein expression levels. The results are presented as mean values ± standard error of the mean (SEM), with statistical significance indicated at a *p*-value below 0.05 (** *p* < 0.01).

This study investigated the role of PPARγ activation in AA-mediated cellular inflammation. As shown in Figure 12, GW9662 inhibited PPARγ while also preventing the inhibition of NF-κB activation by AA. These findings highlight the role of AA as a PPARγ activator in LPS-stimulated RAW264.7 cells.

## 3. Discussion

Multiple studies have shown that AA has anti-hyperlipidemic, anti-inflammatory, and antioxidant effects, demonstrating its potential use as an anti-atherosclerotic agent. To further study the potential mechanism of AA against AS, PPI network analysis was performed to predict the potential targets. Then, the core targets in the network (TNF, IL-6, PPARγ, PTGS2, and IL-1β) were obtained according to the degree value. The TNF superfamily plays a crucial role in cardiovascular inflammation. TNF-α has an important effect on the advancement of endothelial dysfunction and atherosclerosis [33]. IL-6 is believed to be involved in promoting the occurrence and progression of atherosclerosis through multiple pathways. Research has demonstrated that measuring IL-6 levels in plasma can provide an indicator of the presence of cardiovascular disease [34]. PPARγ is recognized as a protective factor against the pathogenesis of AS. It plays a key role in regulating lipid uptake, transformation, and clearance, as well as inhibiting inflammatory responses [35]. PTGS2, also referred to as COX-2, is a significant factor in the regulation of AS progression, with its expression level being directly linked to the severity of AS [36]. IL-1β is capable of triggering inflammatory responses in endothelial cells and plays a role in various phases of AS [37].

GO enrichment analysis revealed that regulating the inflammatory response to lipopolysaccharide plays a crucial role in the treatment of AS. LPS is commonly found in the outer membrane of Gram-negative bacteria. Research indicates that LPS can trigger inflammatory responses in AS by activating inflammatory cells such as macrophages and monocytes [38]. Epidemiological evidence suggests that subclinical levels of LPS may increase the risk of developing atherosclerosis [39]. Therefore, controlling LPS-induced inflammatory responses could be a key strategy in managing AS. Additionally, the pathways identified through KEGG analysis included those involved in the IL-17, TNF, and PPAR signaling pathways, among others. These pathways are mainly related to physiological effects such as anti-inflammatory and antioxidant stress and the regulation of lipid metabolism. 

The five core targets identified using the cytoHuba plug-in were utilized for molecular docking. The results revealed that AA demonstrated significant interactions with TNF, IL-6, PPARγ, PTGS2, and IL-1β, among which it has the lowest binding energy with PPARγ. MD simulations further confirmed the docking results, and the AA-PPARγ protein complex showed good stability in terms of RMSD, RMSF, Rg, SASA, and Gibbs FEL. AA forms many hydrogen bond interactions with important residues shared by PPARγ partial agonists, including Ser289 and Cys285 [40]. Studies have shown that AA can improve cardiac function and reduce the size of myocardial infarction in rats after myocardial ischemia–reperfusion. This may be achieved by upregulating the expression of PPARγ in the ischemic myocardium to enhance anti-inflammatory and antioxidant effects [41]. In addition, AA can effectively reduce liver I/R injury through the PPARγ/NLRP3 inflammasome signaling pathway. PPARγ antagonist (GW9662) abolishes the hepatoprotective effect of AA [42].

RAW264.7 cells are considered a suitable model for studying macrophages. Under LPS stimulation, RAW264.7 cells increased NO production and enhanced phagocytosis. Inhibiting the production of NO is crucial in treating inflammatory diseases [43]. The LPS-induced macrophage model is widely used to screen drugs with anti-inflammatory and anti-atherosclerotic effects [44]. The results showed that the significant increase in NO levels induced by LPS stimulation could be inhibited by AA in a dose-dependent manner. Previous research has demonstrated that LPS stimulation enhances ROS production in macrophages, which in turn acts as a secondary messenger to regulate the expression of pro-inflammatory genes [45]. This study showed that AA significantly reduced the accumulation of ROS in RAW264.7 cells treated with LPS. AA was observed to suppress the expression of pro-inflammatory cytokines, including TNF-α, IL-6, iNOS, and COX-2. Interestingly, AA was also found to upregulate the expression of Arg-1, a marker for M2 macrophages. These results indicate that AA not only suppresses pro-inflammatory cytokines but also promotes M2 macrophage polarization.

This study further studied the effect of AA on LPS-induced inflammatory signaling pathways to explore its molecular mechanisms. NF-κB has been identified as a key upstream signal in the inflammatory cascade, and by inhibiting NF-κB, the expression of inflammatory factors can be reduced [46]. Previous research has demonstrated that the PPARγ/NF-κB signaling pathway plays a significant role in regulating the inflammatory response [47]. In this study, AA was found to reverse LPS-induced NF-κB activation by upregulating PPARγ protein expression and inhibiting NF-κB p65 phosphorylation. Treatment with the PPARγ-specific inhibitor GW9662 inhibited PPARγ expression, and LPS-induced NF-κB activation could also be reversed. Therefore, it is hypothesized that the PPARγ/NF-κB signaling pathway could be a crucial target for AA in controlling inflammatory responses. Overall, our study sheds light on the potential of and identified promising targets for AA in regulating inflammation in AS, providing insights into its mechanism of action at a systemic level. Our findings suggest that AA may modulate NF-κB/PPARγ signaling and influence macrophage polarization to attenuate the inflammatory response, thereby providing therapeutic benefits for AS. Nevertheless, further validation through animal models is necessary to confirm the pathways and targets identified. 

## 4. Materials and Methods

### 4.1. Identification of Potential Targets of AA

The canonical SMILES of AA was retrieved from the PubChem database (PubChem CID: 119034) and subsequently imported into the HERB, Encyclopedia of Traditional Chinese Medicine (ETCM), and SwissTargetPrediction databases to obtain potential targets. The AS-related targets were collected from the GeneCards database, and the targets with relevance ≥ 1 were selected. All the targets were subsequently standardized using the UniProt protein database.

### 4.2. Protein–Protein Interaction (PPI) Analyses

Common disease and drug targets were identified using venny2.1.0. The protein–protein interaction (PPI) network was constructed using the Search Tool for Interactive Genes (STRING) database (Version 12.0). The species selection was “Homo sapiens” with a confidence score > 0.4. Subsequently, the PPI network was imported into Cytoscape (Version 3.10) to identify the hub genes. The “degree” and “clustering coefficient” are beneficial for topological analysis, and the top-ranked targets were selected as hub targets based on the degree levels. 

### 4.3. Gene Ontology (GO) and Kyoto Encyclopedia of Genes and Genomes (KEGG) Enrichment Analyses

GO and KEGG enrichment analyses of the common targets were performed using the ClusterProfiler R package (Version 3.6.1). KEGG enrichment analysis identifies possible biological pathways and functions associated with targets, whereas GO focuses on the biological process (BP), molecular function (MF), and cellular composition (CC). The screening was based on *p* < 0.05.

### 4.4. Molecular Docking 

The receptor proteins IL1β (PDB ID: 5R7W. https://www.rcsb.org/structure/5R7W (accessed on 2 March 2024)), IL6 (PDB ID: 1ALU [48]), PPARγ (PDB ID: 6MS7 [49]), PTGS2 (PDB ID: 5F1A [50]), and TNF (PDB ID: 7JRA [51]) were obtained from the Protein Data Bank (PDB) database (https://www1.rcsb.org/ (accessed on 2 March 2024)). PyMOL 2.3.0 software was employed to examine the protein structure for subsequent docking procedures. The 3D structures of AA and VSP-77 were downloaded from the PubChem database (https://pubchem.ncbi.nlm.nih.gov (accessed on 18 June 2024)). The conformation of AA was optimized using the MMFF94 force field in OpenBabel 3.1.1 software, thereby achieving the lowest energy state of the optimal molecular structure [52]. Hydrogenation of proteins was performed using AutoDock Tools 1.5.6, along with the hydrogenation and determination of rotatable bonds for AA [53]. Then, the structures were saved as pdbqt files. A semi-flexible docking method was used, with an exhaustiveness setting of 25 for accuracy. The Lamarckian genetic algorithm was set as the docking algorithm.

The parameters for molecular docking were configured in the grid section, and the docking parameters were set as follows: TNF (enter_x = −11.1, center_y = −1.7, center_z = −28.1, size_x = 40, size_y = 40, and size_z = 40). IL6 (enter_x = 6.1, center_y = −27.4, center_z = 8.8, size_x = 40, size_y = 40, and size_z = 40). PPARγ (enter_x = 46.6, center_y = 8.9, center_z = 18.6, size_x = 48, size_y = 56, and size_z = 66). PTGS2 (enter_x = 38.4, center_y = 23.2, center_z = 232.6, size_x = 47.3, size_y = 41.3, and size_z = 35.3). IL1β (enter_x = 39.3, center_y = 12.1, center_z = 69.3, size_x = 37.5, size_y = 25.0, and size_z = 37.5). Finally, AutoDock Vina 1.2.0 software was used for molecular docking.

### 4.5. Molecular Dynamics Simulation

Gromacs 2020.6 software was used for the MD simulation of the AA–PPARγ complex and the VSP-77–PPARγ complex. Regarding the force field, Amber14sb was selected for the protein and GAFF2 for the ligand. The SPC/E water model was used to solvate the protein–ligand system. For the water box, a periodic boundary box of 1.2 nm was established. Long-range electrostatic interactions were calculated using the particle mesh Ewald (PME) method, and neutralizing ions were added to the system via Monte Carlo ion placement. Before the formal MD simulation, the complex was energy-minimized using the steepest descent algorithm for 50,000 steps until the maximum force dropped to 1000 kJ/mol. Subsequently, the system was further equilibrated for 100 ps under canonical ensemble (NVT) and isothermal–isobaric ensemble (NPT) conditions at 310 K and 1 atm. Finally, a 100 ns MD simulation with a time step of 2 fs was performed to observe the system changes. The coordinates were saved every 10 ps to generate a trajectory.

### 4.6. Materials and Reagents

AA (HY-N0194) and the PPARγ inhibitor GW9662 (HY-16578) were purchased from MedChemExpress. The RAW264.7 cell line, Dulbecco’s modified Eagle’s medium (DMEM), and fetal bovine serum (FBS) were purchased from Pricella (Wuhan Pricella Biotechnology Co., Ltd., Wuhan, China). LPS (L2880) was obtained from Sigma-Aldrich (St. Louis, MO, USA). The Cell Counting Kit (CCK)-8 assay kit (C0042), NO Assay Kit (S0021S), and ROS Assay Kit (S0033S) were purchased from the Beyotime Institute of Biotechnology, Shanghai, China. Primary antibodies against IL6, iNOS, TNF α, COX-2, PPARγ, pho-NF-κB p65, NF-κB p65, and GAPDH were purchased from Proteintech Group, Inc., Wuhan, China. Arg-1 and α-Tubulin were purchased from HuaBio, Inc., Hangzhou, China. 

### 4.7. Cell Culture

RAW264.7 cells were cultured in DMEM supplemented with penicillin (100 U/mL), streptomycin (100 μg/mL), and 10% FBS. The cells were incubated at 37 °C and 5% CO_2_ in a cell culture incubator. The culture medium was changed every two days.

### 4.8. Cell Viability Assay

The cell suspension (5000 cells/100 μL/well) was seeded into a 96-well plate, inoculated in a 5% CO_2_ incubator, and cultured at 37 °C for 24 h. The cells were treated with different concentrations of AA (5, 10, 20, 40, 60, 80, and 100 µmol/L) for 24 h, then incubated with 10 µL of CCK-8 working solution at 37 °C for 2 h. The absorbance was measured at 450 nm, and cell viability was calculated.

### 4.9. Morphological Evaluation of AA on LPS-Induced RAW264.7 Cells

RAW264.7 cells were plated in 6-well plates at a density of 1 × 10^5^ cells per well and pretreated with AA dissolved in DMSO at concentrations of 10, 20, and 40 μmol/L. After pretreatment with AA for 2 h, the cells were exposed to LPS (1 μg/mL) for 24 h. Cell morphology was observed under an inverted light microscope and photographed.

## 5. Determination of NO Levels in RAW264.7 Cells

Cells were plated in 6-well plates and treated as previously described in Section 4.9. NO production in cell culture media was measured using a total nitric oxide assay kit according to the manufacturer’s protocol. Then, the absorption was measured at 540 nm.

### 5.1. Measurement of Intracellular ROS Generation

The 2′,7′-dichlorodihydrofluorescein diacetate (DCFH-DA) method was used to detect the intracellular ROS production. Cells were incubated with DCFH-DA (1:1000 dilution with serum-free medium) for 30 min at 37 °C in the dark. Then, the cells were washed three times with PBS to remove the DCFH-DA that had not entered the cells. Further, the fluorescence was immediately observed with an inverted fluorescence microscope at an excitation wavelength of 485 nm and an emission wavelength of 530 nm. Finally, the total green fluorescence intensity of each well was quantitatively analyzed using image analysis software.

### 5.2. Western Blotting

Total cellular proteins were collected and quantified, and then a protein loading buffer was added and boiled for degeneration. Protein samples were separated using 10% SDS-PAGE gel and transferred to polyvinylidene fluoride (PVDF) membranes. The membranes were blocked in 5% non-fat milk with Tween-20 buffer for 1 h, followed by overnight incubation with primary antibodies at 4 °C. Subsequently, incubation with secondary antibodies was performed for 1 h at room temperature before ECL detection was carried out. Relative protein expression was quantified using Image-J software (Version 1.54).

### 5.3. Statistical Methods

Statistical analysis was performed using GraphPad Prism 9 (Version 9.5.0). Normal distribution was tested using the Kolmogorov–Smirnov or Shapiro–Wilk test. Student’s t-test was performed to compare two groups, and a one-way analysis of variance (ANOVA) was performed to test three or more groups. The results were expressed as mean ± SEM. *p* < 0.05 was considered statistically significant.

## 6. Conclusions

This study utilized network pharmacology and experimental validation to investigate the pharmacological activity mechanism of the natural compound AA and its potential in treating AS. The findings indicate that AA primarily targets key molecules such as TNF, IL-6, PPARγ, PTGS2, and IL-1β and reduces inflammation by modulating the PPARγ/NF-κB signaling pathway, as confirmed through cell experiments. As a result, this research conducted an initial exploration of the anti-atherosclerotic mechanism of AA through network pharmacology and experimental validation, laying the groundwork for future animal studies.

## Data Availability

The original contributions presented in the study are included in the article, further inquiries can be directed to the corresponding authors.
